# Salicylate-Induced Changes in Hearing Thresholds in Mongolian Gerbils Are Correlated With Tinnitus Frequency but Not With Tinnitus Strength

**DOI:** 10.3389/fnbeh.2021.698516

**Published:** 2021-07-30

**Authors:** Veralice Lanaia, Konstantin Tziridis, Holger Schulze

**Affiliations:** Experimental Otolaryngology, University Hospital Erlangen, Friedrich–Alexander University Erlangen–Nürnberg, Erlangen, Germany

**Keywords:** tinnitus, effect size, salicylate, noise trauma, stochastic resonance, hearing threshold

## Abstract

Tinnitus is an auditory phantom percept without external sound sources. Despite the high prevalence and tinnitus-associated distress of affected patients, the pathophysiology of tinnitus remains largely unknown, making prevention and treatments difficult to develop. In order to elucidate the pathophysiology of tinnitus, animal models are used where tinnitus is induced either permanently by noise trauma or transiently by the application of salicylate. In a model of trauma-induced tinnitus, we have suggested a central origin of tinnitus-related development of neuronal hyperactivity based on stochastic resonance (SR). SR refers to the physiological phenomenon that weak subthreshold signals for given sensors (or synapses) can still be detected and transmitted if appropriate noise is added to the input of the sensor. The main objective of this study was to characterize the neurophysiological and behavioral effects during salicylate-induced tinnitus and compare these to the conditions within the trauma model. Our data show, in line with the pharmacokinetics, that hearing thresholds generally increase 2 h after salicylate injections. This increase was significantly stronger within the region of best hearing compared to other frequencies. Furthermore, animals showed behavioral signs of tinnitus during that time window and frequency range as assessed by gap prepulse inhibition of the acoustic startle reflex (GPIAS). In contrast to animals with noise trauma-induced tinnitus, salicylate-induced tinnitus animals showed no correlation between hearing thresholds and behavioral signs of tinnitus, indicating that the development of tinnitus after salicylate injection is not based on SR as proposed for the trauma model. In other words, salicylate-induced tinnitus and noise trauma-induced tinnitus are not based on the same neurophysiological mechanism.

## Introduction

Diseases of the inner ear that lead to hearing loss (HL) may also result in subjective tinnitus (Ahlf et al., 2012), an auditory phantom sensation that is experienced, although no physical sound is present. Tinnitus occurs with surprisingly high prevalence, affecting about 35% (Shargorodsky et al., 2010) of the general population, with 10–15% of individuals experiencing prolonged periods of tinnitus that require medical evaluation. For 10% of the population, tinnitus has a significant impact on their quality of life (Chao et al., 2014). Despite this high prevalence and the tinnitus-associated distress of affected patients, which, in severe cases, may experience insomnia, psychological disorders like depression, the inability to work, or even commit suicide (Coles, 1984; Lewis et al., 1994; Langguth et al., 2011), the cause(s) and pathophysiology of tinnitus are still controversially discussed, making prevention and treatments difficult to develop (Turner et al., 2006). More than 20 years ago, tinnitus was thought to result from aberrant neural activity generated in the periphery of the auditory system (Jastreboff, 1990). In particular, tinnitus was proposed to result from increased activity in the cochlear nerve. More recently, due to the developments in basic neuroscience, a central origin of tinnitus-related activity seems to have replaced the former peripheral hypothesis (Noreña and Farley, 2013). The main reason for this shift is based on evidence that surgical neurotomy of the cochlear nerve, which should suppress tinnitus if the activity driving the percept originated in the cochlear nerve, has hardly ever had this intended effect (House and Brackman, 1981; Barrs and Brackmann, 1984; Silverstein et al., 1986; Pulec, 1995; Baguley et al., 2002). An effective cure for tinnitus still does not exist, and the main reason is that the neurophysiological mechanism that leads to the development of tinnitus is still not fully understood. Until today, several tinnitus models are being debated, although, due to developments in basic neuroscience, a central origin of tinnitus-related activity seems to have replaced the former peripheral hypothesis (Noreña and Farley, 2013). In particular, three main models, which are based on altered lateral inhibition, homeostatic plasticity, or stochastic resonance (SR) (Gerken, 1996; Eggermont and Roberts, 2004; Schaette and McAlpine, 2011; Ahlf et al., 2012; Tziridis et al., 2015; Leaver et al., 2016; Vanneste and De Ridder, 2016; Schilling et al., 2020), propose a central origin of tinnitus, resulting from damage to the cochlea as the initial step in tinnitus development.

In our recent work, Krauss and colleagues (Krauss et al., 2016) have suggested a central origin of tinnitus-related development of neuronal hyperactivity based on SR, which refers to the phenomenon that weak signals, which are subthreshold for a given sensor (or synapse), can still be detected and transmitted by that sensor if (neuronal) noise is added to the sensor input. We assumed that SR at the level of the dorsal cochlear nucleus constantly optimizes information transmission into the auditory system and, thereby, may, e.g., compensate for hearing loss. In this view, the noise necessary for SR is then the neurophysiological source of tinnitus-related enhanced neuronal activity.

The two main tinnitus inducers in humans are noise trauma (Chermak and Dengerink, 1987; Metternich and Brusis, 1999; Temmel et al., 1999; Stankiewicz et al., 2000; Mrena et al., 2002; Langguth et al., 2011) and high dose of salicylate (Myers and Bernstein, 1965; McFadden et al., 1984; Day et al., 1989; Cazals, 2000; Baguley et al., 2002; Langguth et al., 2011). In this study, the effect of salicylate is tested in Mongolian gerbils, and the results are compared to data of noise trauma-induced tinnitus (Ahlf et al., 2012; Tziridis et al., 2015) to investigate if those two types of tinnitus are based on the same neurophysiological mechanism or not.

We induce tinnitus in Mongolian gerbils, because, in contrast to mice and rats, the hearing of the gerbil up to 20 kHz closely resembles the human audiogram (best hearing around 4 kHz) (Ryan, 1976). We, here, compare neurophysiological and behavioral markers of tinnitus in animals receiving a noise trauma at 2 kHz and 115 dB SPL for 75 min and animals receiving subcutaneous injections of a high dose of salicylate (300 mg/kg), as it has been demonstrated before that salicylate doses between 150 and 350 mg/kg induce tinnitus in rodents (Sheppard et al., 2014). Behavioral estimates of salicylate-and noise trauma-induced tinnitus were obtained, using gap prepulse inhibition of the acoustic startle reflex (GPIAS, cf. Schilling et al., 2017), and auditory brainstem responses (ABR) were recorded to monitor changes in central auditory activity. We tested frequency-specific differences in GPIAS after noise trauma and salicylate treatment, and we correlated these data with possible ABR threshold changes in the same animals to evaluate similarities and differences of both tinnitus models. Taken together, the aim of the study was the characterization of neurophysiological and behavioral effects of salicylate-induced tinnitus and its comparison with data obtained with the noise trauma-induced tinnitus in the context of our model of SR-based tinnitus development.

## Materials and Methods

### Ethics Statement and Animals

Mongolian gerbils (*Meriones unguiculatus*) were housed in standard animal racks (Bio A. S. Vent Light, EHRET Labor- und Pharmatechnik, Emmendingen, Germany) in groups of 2–3 animals per cage with free access to water and food at 20–24°C room temperature under a 12/12-h dark/light cycle. The use and care of animals were approved by the state of Bavaria (reference No. 55.2-2532-2-726, Regierungspräsidium Unterfranken, Würzburg, Germany).

### Salicylate Treatment

**FIGURE 1:**
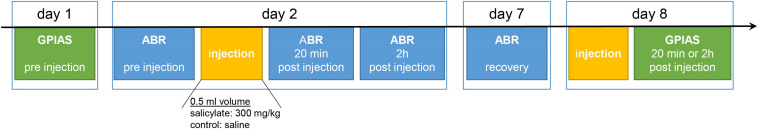
Timeline of experiments. We first measured the baseline behavior in the prepulse inhibition (PPI) of the acoustic startle reflex (ASR) of each animal. The next day, we measured the audiograms, using pure tone ABR (pure tone hearing threshold, HT), first before the injection and, subsequently, 20 min and 2 h after the injection in both groups. After 7 days, we, again, measured the audiograms of the same animals. On the 8th day, we proceeded with a second injection of either salicylate or saline in the same (now awake) animals and obtained the 20-min or 2-h behavior response in the PPI of the ASR.

A total number of 37 10-week-old male gerbils purchased from Janvier (Saint Berthevin Cedex, France) were used in this study. Eighteen animals were treated with subcutaneous injection of isotonic saline (control group C, ∼0.5 ml) and 19 animals with subcutaneous injection of sodium salicylate (group S, 300 mg/kg; Sigma), dissolved in the saline, resulting in the same amount of injection volume (∼0.5 ml). All animals were examined, using the GPIAS and ABR measurements (Figure 1). We first measured the baseline behavior in the prepulse inhibition (PPI) of the acoustic startle reflex (ASR) of each animal (cf. below). The next day, we measured the audiograms, using pure tone ABR (pure tone hearing threshold, HT), first before the injection and, subsequently, 20 min and 2 h after the injection in both groups (cf. below). After 7 days, we, again, measured the audiograms of the animals in order to evaluate possible long-term effects of the salicylate treatment on HT. Once it was certain that the effect had disappeared, we proceeded with a second injection of either salicylate or saline in the same (now awake) animals and obtained the GPIAS again to assess a possible acute tinnitus percept. Control and salicylate animals were separated into two groups based on the temporal delay of the behavioral test after the injection. Nine animals of the control group were behaviorally tested 20 min after the injection, and another nine control animals were behaviorally tested 2 h post-injection. In the salicylate group, 10 animals were tested 20 min, following the injection and, again, 2 h, following the injection, and nine animals were behaviorally tested 2 h post-injection only. There was no significant difference (*t*-tests with *p*-value between 0.14 and 0.7); the 2-h responses of these animals and were, therefore, treated as one 2-h group. We later analyzed frequency-specific differences in the startle reflex responses after injection and correlated these data with post vs. pre-injection differences in the ABR thresholds (cf. below).

### Data of Animals Treated With Acoustic Trauma

For the comparison of the salicylate with trauma data, we reanalyzed GPIAS and ABR threshold data of 16 animals treated with a binaural acoustic noise trauma of 2 kHz and 115 dB SPL over 75 min under anesthesia. All methods of data recording are already published (Ahlf et al., 2012; Tziridis et al., 2015). In a nutshell, the trauma for tinnitus induction was applied under deep ketamine-xylazine anesthesia as described in detail earlier (Ahlf et al., 2012; Walter et al., 2012; Tziridis et al., 2014, 2015). The anesthetized animals were placed on a heating pad with a remote-controlled temperature of 37°C, centered in front of a loudspeaker (Canton Plus X Series 2; Canton, Weilrod, Germany). Using a signal generator (hp 33120A, HP, Böblingen, Germany) connected to an audio amplifier (Amp 75, ThomasWulf, Frankfurt, Germany), a 2 kHz pure tone was presented at a sound pressure level of 115 dB SPL for 75 min.

Pre GPIAS and ABR recordings were performed during the week prior to the trauma (cf. Figure 1). The post-trauma ABR was recorded during a 2-h period after the treatment when trauma effects were strongest. The behavioral responses were recorded 5 to 7 days after the trauma when the animals completely recovered from the procedure and a possible tinnitus percept reached its subacute phase. Datasets were analyzed with our improved methods of GPIAS (Schilling et al., 2017) and ABR threshold evaluation (Schilling et al., 2019) (cf. below). Data of both measurements were correlated with each other in the same way as in the salicylate/saline animals.

### Behavioral and ABR Measurements

All methods used in this paper have been described previously (Tziridis et al., 2015; Schilling et al., 2017, 2019) but will be explained shortly here for better intelligibility.

### Auditory Brainstem Response

As described by Schilling and coworkers (Schilling et al., 2019), ABR measurements were recorded, using a custom-made setup. Pure tone stimuli of different frequencies, ranging from 1 to 8 kHz, were generated by a custom-made Python program (Python 3.6.0 and presented at different intensities, ranging from 30 to 90 dB SPL in 5 dB steps. Stimulation was performed free-field *via* a speaker (Sinus Live NEO), corrected for its frequency transfer function to be flat within ±1 dB at a distance of ∼ 3 cm from the pinna of the animal. To compensate for speaker artifacts, stimuli were presented in double trials, consisting of two 6-ms stimuli (including 2-ms sine square rise and fall ramps) of the same amplitude but an opposite phase, separated by 100 ms of silence. A number of 250 trials of each combination of intensity and frequency were presented pseudorandomly at an interstimulus interval of 500 ms. Mongolian gerbils were anesthetized with a mixture of ketamine (Ketaset 100 mg/ml) and medetomidine (Dorbene 1 mg/ml) (mixture of ketamine 75 mg/kg BW; medetomidine 0.5 mg/kg BW; atropine sulfate 0.3 mg/kg BW in saline. Deep anesthesia was ensured by an initial subcutaneous injection of 0.4 ml of the anesthetic solution and maintained by application of 0.1 ml/h. During measurements, the animals were placed on a feedback-controlled heating pad at 37°C to maintain body temperature. Data were recorded, using three silver electrodes positioned subcutaneously, one for grounding at the back of the animals, one reference electrode at the forehead, and the measuring electrode infra-auricular, overlying the bulla of the recording side of the left ear. The potential difference between reference and measuring electrode was amplified by a low-noise amplifier (JHM NeuroAmp 401, J. Helbig Messtechnik, Mainaschaff, Germany; amplification 10,000; bandpass filter 400 to 2,000 Hz and 50 Hz notch filter). The output signal of the amplifier was digitalized and recorded by an analog-digital converter card (National Instruments Corporation, Austin, TX, United States) with a sampling rate of 20 kHz and synchronized with the stimulation *via* the trigger signal from the stimulation computer. Raw data of 250 double trials per sound level for each stimulus frequency were averaged. Finally, these averaged responses of the two single-phase-inverted stimuli within one double trial were averaged to eliminate stimulus artifacts. From this average, artifact-corrected data, the root mean square (RMS) amplitude values from 0 to 10 ms after the stimulus onset were calculated to obtain a measure of response strength for each stimulus presented. The HT of the animals was automatically estimated before and after the injection of salicylate or saline (Schilling et al., 2019). Furthermore, the hearing loss (HL), i.e., the difference between the HT values after the injection minus the values of the threshold before the injection was calculated.

### Behavioral Assessment of Tinnitus

As described by Schilling and coworkers (Schilling et al., 2017), the animals were placed in a transparent acrylic tube (length, 10 cm; inner diameter, 4.3 cm), which was positioned at a distance of 10 cm in front of a loudspeaker (Canton Plus X Series 2), on a low-vibration table (TMC, Peabody, MA, United States). The whole setup was placed in an acoustic chamber (Industrial Acoustics Company GmbH, Niederkrüchten, Germany). The startle response was measured by a sensor platform with three integrated acceleration sensors (ADXL 335 on GY 61 board, Robotpark). All calibration measurements were made in the restrainer to correct for acoustical perturbations. The front end of the tube was closed with a stainless steel grate (wire mesh, width 0.5 mm), allowing for acoustic stimulation with no detectable distortion within the used frequency range of stimulation (a signal-to-noise ratio of at least 70 dB). Sound pressure level (SPL) was calibrated, using a condenser microphone (Brüel and Kjaer Type 4190) *via* a preamplifier (Brüel and Kjaer Type 2669) and a measuring amplifier (Brüel and Kjaer Type 2610). Stimulus generation and data acquisition used custom-made programs (Python, Version 3.6.0) (Gerum et al., 2019). As startle amplitudes tend to be higher for the first few trials, five startle stimuli were presented before the beginning of each measurement to rule out strong habituation effects. For sound generation, the frequency response function of the speaker was calibrated to produce an output spectrum that was flat within ± 1 dB. The animals were placed in the tube, in which they fit well and were able to move back and forth roughly 2 cm. We had allowed 10-min habituation time before the GPIAS paradigm started (Turner et al., 2006).

Gap prepulse inhibition of the acoustic startle reflex was used to assess the possible existence of a tinnitus percept and to give a rough estimate of the perceived tinnitus frequencies. The animals were subjected to continuous band pass-filtered 60 dB SPL loud background noise (2-ms cosine square rise and fall times) with medium frequencies of 1, 2, 4, or 8 kHz and a bandwidth of ± half an octave. The mean duration of the background noise before the startle noise burst was 10 ± 2.5 s; it ended at the beginning of the startle noise burst. The startle white noise burst (115 dB SPL, 20 ms) was presented either 50 ms after a 50-ms-long silent gap (2-ms cosine square rise and fall times) in the background noise, or it was presented without any gap. The twitching response of animals to the startle stimulus was recorded as described above. For each background frequency, 40 repetitions with and without gap were presented in randomized order. A single session of the GPIAS experiment took roughly 50 min. Every animal was at least tested two times, the first time before any treatment and the second time either 20 min and/or 2 h after treatment (cf. above).

The analysis of the behavioral data was performed as described by Schilling and coworkers (Schilling et al., 2017). As the response amplitudes of the PPI of the ASR are not normally distributed, the data were first log-normalized. Then, we exploited the full combinatorial power of all normalized response amplitudes to obtain the PPI distributions before and after manipulation of the animals and calculated the effect size of the behavioral response. Positive values indicate a stronger effect of the gap in the post compared with the precondition. Negative values indicate less effect of the gap after treatment, i.e., a stronger startle response despite the present gap, which is considered to indicate a “filling” of the gap by a tinnitus percept in that frequency range. Additionally, these now normally distributed data could be analyzed, using parametrical statistics, like Student’s *T*-test for comparisons of mean changes, and, therefore, statistically significant changes of the effect size of the PPI change can be used to define the strength of a possible tinnitus percept represented by negative effect size values.

### Statistical Analysis

For statistical analysis, we used Statistica 8 (StatSoft Hamburg, Germany). We performed one-factorial repeated measurement mixed ANOVAs for the variables HT, HL, and effect size over the presented *frequencies* with the repetition factor *time relative to injection* for salicylate and the control group separately. Tukey’s *post hoc* test was used to further assess the differences in the data. For the comparison between the control and salicylate groups, we used two-factorial ANOVAs with the factors *group* and *frequency* at the three different time points independently. Again, Tukey’s *post hoc* tests were used to further asses the differences in the data. We also investigated the correlations between the effect size and HL by multiple linear regression analyses to assess the underlying neurophysiological dependencies of electrophysiology and behavior.

## Results

### Effects of Salicylate Injection on ABR Thresholds

#### The Hearing Threshold in the Control Group

**FIGURE 2:**
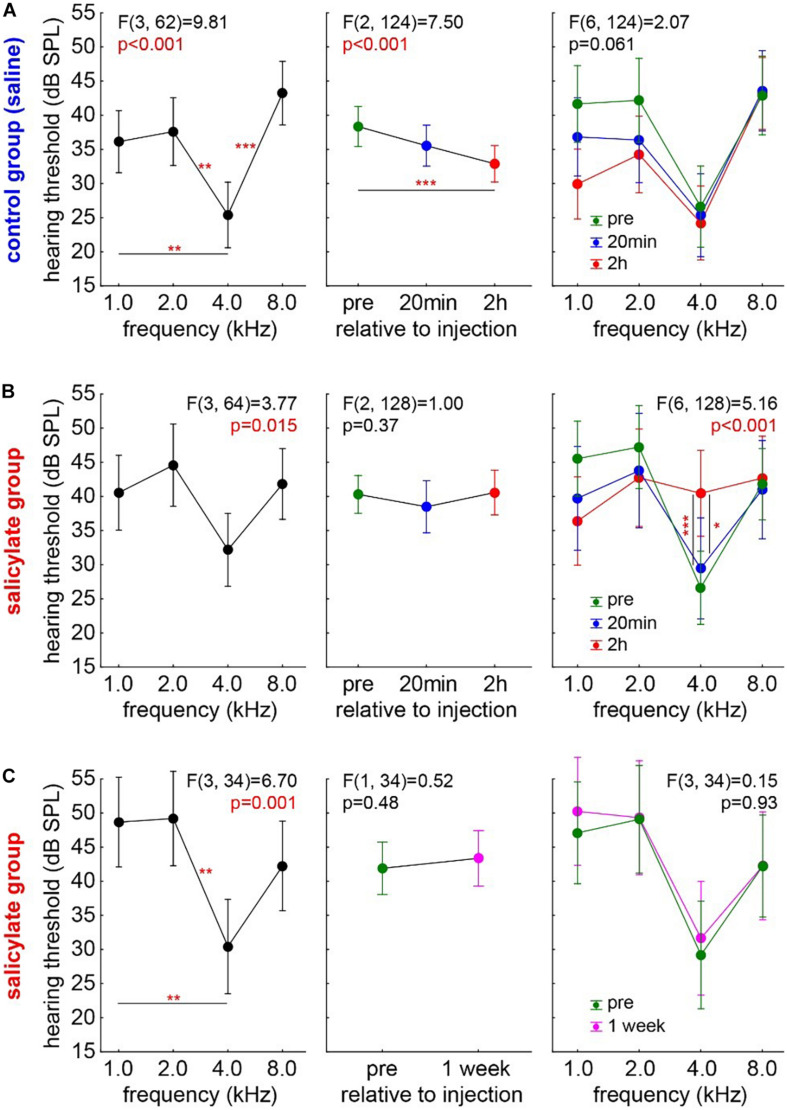
Development of HT over time in both experimental animal groups. One-factorial repeated measurement mixed ANOVA with the factor *frequency* (left panels) and the repetition factor *time* (center panels) and interaction of *time X frequency* (right panels) are shown: **(A)** Audiogram in the control group (group C, *n* = 18). Over time, a significant decrease of the frequency-averaged hearing threshold can be observed. No interaction between *frequency* and *time* was found. Asterisks indicate a level of Tukey *post hoc* tests: *p < 0.05, **p < 0.01, ***p < 0.001. **(B)** Audiogram in the salicylate group (group S, *n* = 19). No HT difference over time was found, but a significant interaction of *time X frequency* was observed with the strongest effect centered around 4 kHz. **(C)** The transitory effect of the salicylate. No HT differences in the same salicylate animals 1 week post-injection compared with their pre injection recordings.

First, the audiograms of 18 control animals (group C) were measured. In detail, the results of a one-factorial repeated measurement mixed ANOVA with the factor *frequency* and the repetition factor *time* and interaction of *time X frequency* are given in Figure 2A. The Figure 2A left panel shows the mean audiogram (*factor frequency*) averaged across the different time points in control gerbils, with the best hearing frequency at 4 kHz. Over *time* (Figure 2A center panel), we observed a significant decrease of the frequency-averaged hearing threshold (mean pre ± standard deviation: 38.45 ± 13.45 dB SPL; mean 20 min post saline injection: 35.68 ± 13.56 dB SPL; mean, 2 h post saline injection: 32.94 ± 12.72 dB SPL). *Post hoc* Tukey tests showed *p* < 0.001 between pre and 2 h post saline injection HT, while pre vs. 20 min post-injection and 2 h vs. 20 min post-injection HT were not significantly different. Furthermore, there was no interaction between *frequency* and *time* [Figure 2A, right panel; *F*(6,124) = 2.07, *p* = 0.06], indicating that the HT difference over time, possibly induced by anesthetics, was not frequencies dependent. In conclusion, the animals present a standard audiogram, and any change in the HT during the 2-h anesthesia may be due to the effects of the anesthetics themselves.

#### The Hearing Threshold in the Salicylate Group

The audiograms in the salicylate group (group S, *n* = 19) were analyzed accordingly (Figure 2B) to find any effects of salicylate on the HT over time. Again, a one-factorial repeated measurement mixed ANOVA with the factor *frequency* and the repetition factor *time* and interaction of *time X frequency* was calculated. In Figure 2B, left panel, the mean audiogram (factor *frequency*) over time is depicted. Figure 2B, center panel, shows the frequency-averaged HT over *time* in gerbils with salicylate injection. In this case, no HT difference over time was found (mean pre: 39.83 ± 13.82 dB SPL; mean, 20 min post SS injection: 38.19 ± 16.31 dB SPL; mean, 2 h post SS injection: 40.53 ± 13.31 dB SPL), pointing to a possible effect of the salicylate that counteracts the threshold-reducing effect observed in the control group. In line with this interpretation, we observed a significant interaction of *time X frequency* [*F*(6,128) = 5.15, *p* < 0.001]. With Tukey *post hoc* tests revealing no difference of 20 min post salicylate injection HT compared with pre-injection HT, but a significant HT increase at 4 kHz 2 h post-injection compared with 20 min (*p* = 0.018) and pre-injection (*p* < 0.001). In other words, compared with the control group, no general HT improvement was found in the salicylate animals, but the contrary effect, i.e., a hearing loss, at the best hearing frequency was identified 2 h after the injection. Nevertheless, the described effect of the salicylate is transitory, as shown in Figure 2C. The one-factorial repeated measurement mixed ANOVA with the factor *frequency* and the repetition factor *time* and interaction of *time X frequency* did not reveal any HT differences in the same S group animals 1 week post-injection compared with their pre-injection recordings of the HT.

#### Comparison of HT Between the Salicylate and the Control Groups

**FIGURE 3:**
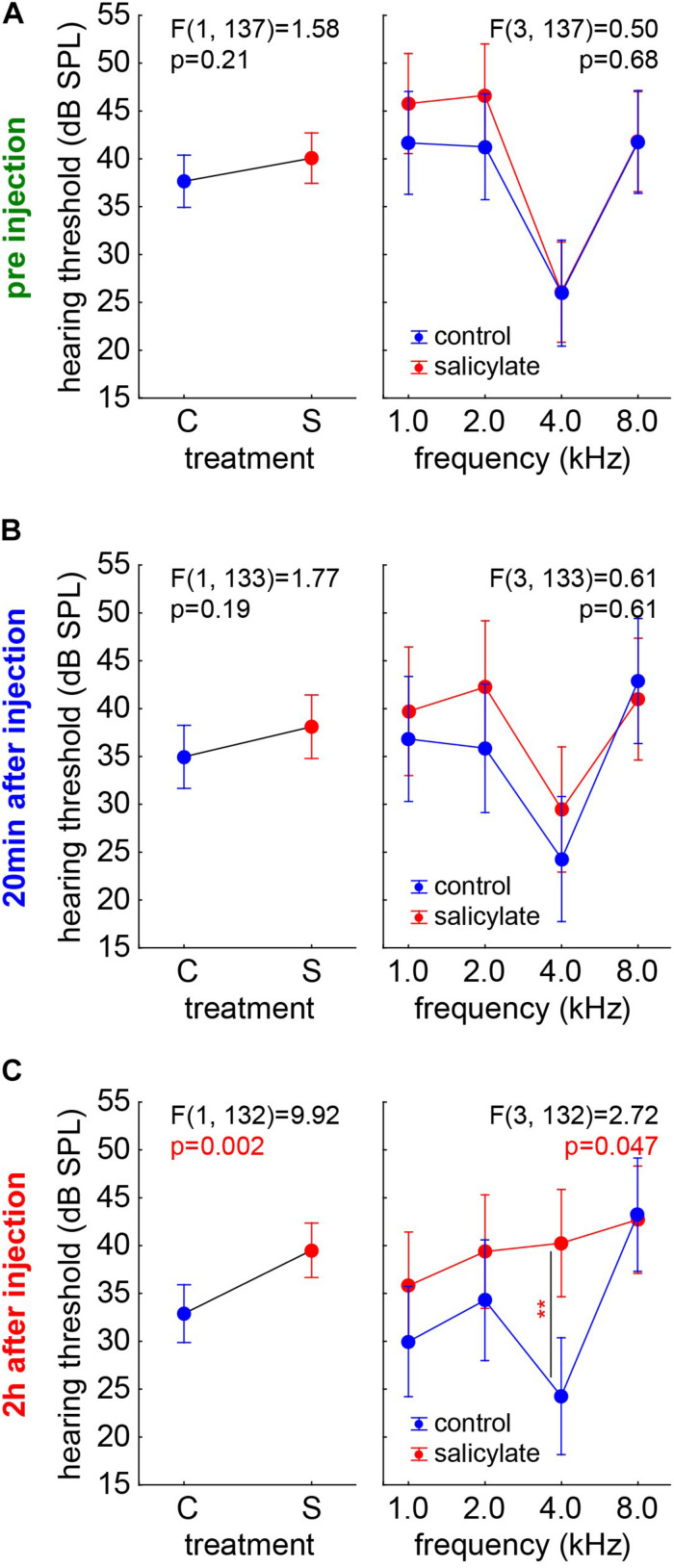
Comparison of the HT of the salicylate and control groups over time. Results of the two-factorial ANOVAs with the factors *group* (left panels) and their interaction with *frequency* (right panels) at the three different time points. **(A)** Before the injection, both groups did not show significantly different HT and no significant interaction of *time X frequency*. **(B)** 20 min after injection, there is no significant different HT, and there is no significant interaction between the factors. **(C)** 2 h after injection, a significant higher HT in the salicylate *group* compared with the control animals and a significant interaction of both factors was found. At 4 kHz, the HT was affected strongest, as indicated by a significant Tukey *post hoc* test (*p* = 0.003).

We compared the salicylate effect on the HT with the possible anesthetics effects in group C by two-factorial ANOVAs with the factors *group* and *frequency* and their interaction at the three different time points independently (Figure 3). While HT showed the typical frequency dependence [factor *frequency*; *F*(3,137) = 20.08, *p* < 0.001], both *groups* did not show significantly different HT before the injection (C: 37.77 ± 13.50 dB SPL; S: 39.98 ± 13.72 dB SPL), and no significant interaction of *time X frequency* emerged (Figure 3A). The same was true at 20 min after the injection [factor *frequency*; *F*(3,133) = 8.08, *p* < 0.001], no difference between mean HT of both groups (C: C: 34.95 ± 13.75 dB SPL; S: 38 ± 16.12 dB SPL) and no significant interaction between the factors (Figure 3B). Nevertheless, after 2 h (Figure 3C, left panel) there was a significantly higher mean HT in the salicylate *group* compared with the control animals [C: 32.94 ± 12.72 dB SPL; S: 39.51 ± 13.53 dB SPL, *F*(1,132) = 9.92, *p* = 0.002], and a significant interaction of both factors [*F*(3,132) = 2.72, *p* = 0.047]. At 4 kHz (which represents the frequency range of best hearing in Mongolian gerbils), the HT was affected strongest, as indicated by a significant Tukey *post hoc* test (*p* = 0.003, Figure 3C, right panel). These data clearly showed no difference in the HT of the animals before the injection of salicylate. Over time, the drug showed its effect with 20 min post-injection, the HT in both groups still being comparable, but, 2 h post injection, the HT of the salicylate group increased specifically at the best hearing frequency of the animals.

#### Hearing Loss in the Control Group

**FIGURE 4:**
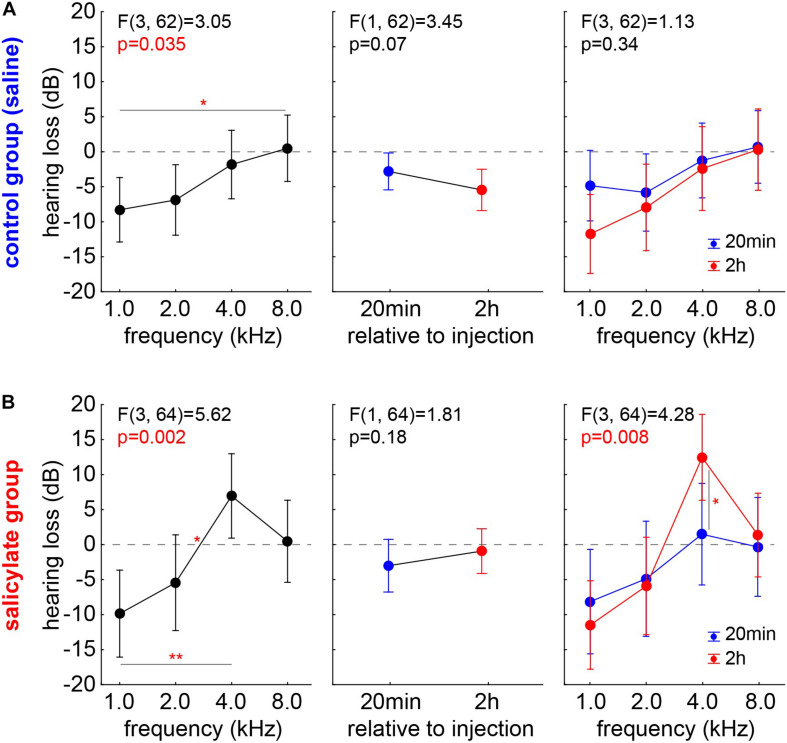
HL in control and salicylate animals analyzed with one factorial repeated measurement mixed ANOVAs with factor *frequency* (left panels), repetition factor *time* (20 min and 2 h; center panels) and the interaction of both factors (right panels). **(A)** In group C, mostly negative HL values could be found over all *frequencies* with significant frequency dependency but no significant difference between the average HL of 20 min and 2 h or in the interaction of the factors could be found. **(B)** In group S, significant *frequency* dependency of the HL was found, no significant HL difference between the two *time* points but a significant interaction of *time X frequency* with a significant Tukey *post hoc* test at 4 kHz emerged. *p < 0.05; **p < 0.01.

To rule out any effect biases of single individuals, we reanalyzed the data, using not the HT but the HL (HT_post_ – HT_pre_), where positive values indicate worse HT_post_, negative values better HT_post_ compared with the pre measurements. As described above, we first analyzed control and salicylate animals with one factorial repeated measurement mixed ANOVAs with factor *frequency*, repetition factor *time* (20 min and 2 h) and the interaction of both factors (Figure 4). In the group C (Figure 4A), mostly negative HL values were found across all *frequencies* with a significant frequency dependency (*p* = 0.035) and a significant Tukey *post hoc* test when comparing 1 kHz with 8 kHz (*p* = 0.048). No significant difference between the average HL of 20 min and 2 h was found (*factor time*: mean 20 min post saline injection: −2.76 ± 10.76 dB; mean 2 h post saline injection: −5.51 ± 12.65 dB). This was also true for the interaction of both factors [*F*(3,62) = 1.13, *p* = 0.34], indicating better hearing at both time points, especially in lower frequency ranges. In other words, better hearing (negative HL) after control injections could be found specifically at lower frequencies, but no significant difference over the two time points emerged, indicating a then stable hearing level of the animals.

#### Hearing Loss in the Salicylate Group

In the group S (Figure 4B), a significant *frequency* dependency of the HL was also found (*p* = 0.002) with a significant positive HL at 4 kHz compared with the negative 1 and 2 kHz HL values (Tukey *post hoc* tests, *p* = 0.001 and *p* = 0.041). Again, no significant HL difference between the two *time* points was detected (mean, 20 min post salicylate injection: −2.78 ± 15.55 dB; mean, 2 h post salicylate injection: −0.44 ± 15.68 dB), but a significant interaction of *time X frequency* [*F*(3,64) = 4.28, *p* < 0.05] with a significant Tukey *post hoc* test at 4 kHz (*p* = 0.011) again indicated the strongest HL at the best hearing frequency after 2 h post salicylate injection.

#### Comparison of HL Between the Control and the Salicylate Groups

The comparison of the HL of groups C and S was again performed by two two-factorial ANOVAs with the factors group and frequency independent for both time points (Supplementary Figure 1). After 20 min (Supplementary Figure 1A) neither factor *frequency* [*F*(3,131) = 2.60, *p* = 0.06] nor the factor *group* [C: −2.55 ± 10.99 dB; S: −2.92 ± 15.49 dB, *F*(1,131) = 0.06, *p* = 0.80] showed any significant effect on the HL, which was also true for the interaction of both factors (*p* = 0.81). After 2 h, on the other hand (Supplementary Figure 1B), significant frequency dependence could be identified [*F*(3,132) = 14.11, *p* < 0.001] but no difference between both groups [C: −5.51 ± 12.65 dB; S: −1.35 ± 15.98 dB, *F*(1,132) = 3.42, *p* = 0.07]. Nevertheless, the significant interaction of both factors [*F*(3,132) = 3.20, *p* = 0.025] and the significant Tukey *post hoc* test at 4 kHz (*p* = 0.007) confirms the findings in the HT described above. So, neither 20 min nor 2 h after injection, a general significant effect of the injection on the HL could be identified. Nevertheless, 2 h after injection with salicylate, the HL data showed a specific increase at 4 kHz only.

### Effects of Salicylate Injection on Behavioral Signs of Tinnitus

#### Effect Size in the Control Group

**FIGURE 5:**
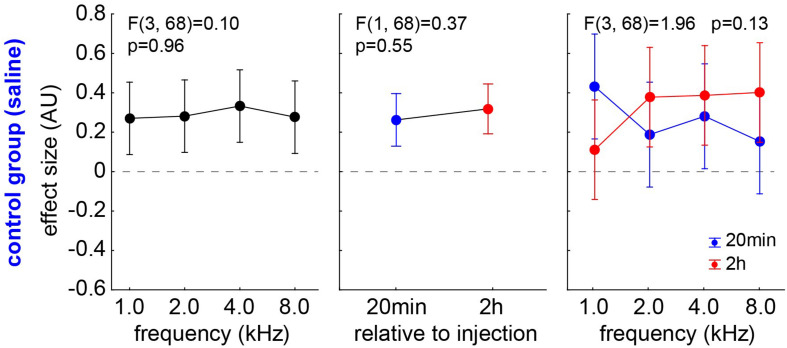
Analysis of effect size (AU) in group C by one-factorial repeated measurement mixed ANOVA with the factor *frequency* and the repetition factor *time*. No significant effect can be found in any of the factors.

Animals of group C showed no behavioral signs of tinnitus in the GPIAS experiments after 20 min post saline injection (*n* = 9; 0/36 *t*-tests with a significant negative effect size) and only in 4/36 cases after 2 h after saline injection (*n* = 9). The chi^2^-test did not show a significant difference between these two time points. Independent of the significance of the effect size, it can be analyzed *via* a one-factorial repeated measurement mixed ANOVA with the factor *frequency* and the repetition factor *time* (Figure 5). No significant effect can be found in any of the factors, i.e., neither in the frequency (*p* = 0.96) nor in the factor time (mean 20 min post saline injection: 0.26 ± 0.32; mean, 2 h post saline injection: 0.31 ± 0.45, *F*(1,68) = 0.37, *p* = 0.55) nor in the interaction (*p* = 0.13). In other words, we only see positive effect sizes that may indicate a cortical learning effect (cf. Discussion).

#### Comparison of the Effect Size Between the Salicylate and the Control Groups

**FIGURE 6:**
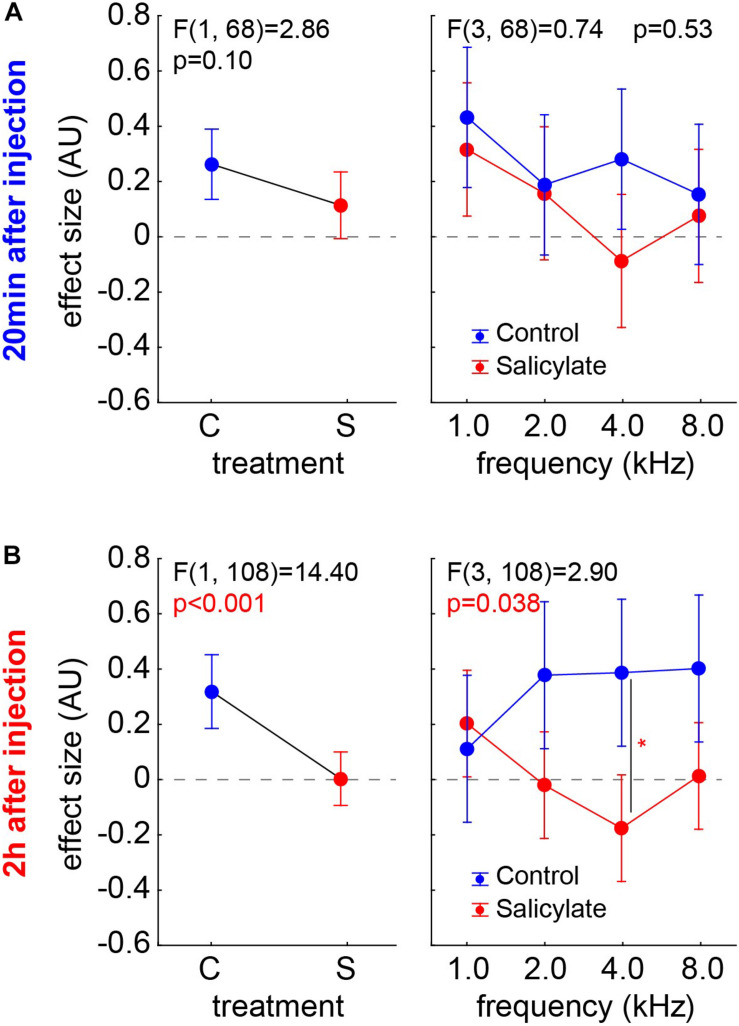
Effect sizes of both animal *groups* (left panels) and their interaction with the factor *frequencies* (right panels) in two-factorial ANOVAs independent for the two time points. **(A)** 20 min after injection (group C, *n* = 9; group S, *n* = 10) – no significant effects are found. **(B)** 2 h after injection (group C, *n* = 9; group S, *n* = 19), the effect size was strongly dependent on the group and also shows a significant interaction, with the Tukey *post hoc* test becoming significant at 4 kHz (*p* = 0.021).

The animals of the S group already started to show first significantly negative effect sizes (*t*-tests, *p* < 0.05) after 20 min after salicylate injection (*n* = 10; 4/40) and doubled that value to eight cases (*n* = 19; 8/69) after 2 h after the injection. Still, the chi^2^-test did not reveal a significant difference between both time points. We compared the effect sizes of both animal *groups* over the different *frequencies* by two two-factorial ANOVAs independently for the two time points (Figure 6). At 20 min after the injection (group C, *n* = 9; group S, *n* = 10) (Figure 6A), no significant effect of *frequency* on the effect size is found [*F*(3,68) = 2.11, *p* = 0.11], which is also true for the effect size comparison across *group* (C:0.26 ± 0.32; S:0.11 ± 0.43; *p* = 0.10) and the interaction of both factors (*p* = 0.53). After 2 h (group C, *n* = 9; group S, *n* = 19), on the other hand (Figure 6B), the effect size still did not depend on the *frequency* [*F*(3,108) = 0.27, *p* = 0.85], but strongly depended on the group [C:0.31 ± 0.45; S:0.003 ± 0.42, *F*(1,108) = 14.40, *p* < 0.001] and also showed a significant interaction [*F*(3,108) = 2.90, *p* = 0.038, with the Tukey *post hoc* test becoming significant at 4 kHz (*p* = 0.021). In other words, after 2 h, we found a significantly lower effect size – with negative values indicating a possible tinnitus percept – in salicylate animals compared with control animals. This difference is most prominent at 4 kHz, which is exactly the same frequency that shows strongest shifts toward higher HT in the ABR.

#### Effects of Noise Trauma on ABR Thresholds and Behavioral Signs of Tinnitus

**FIGURE 7:**
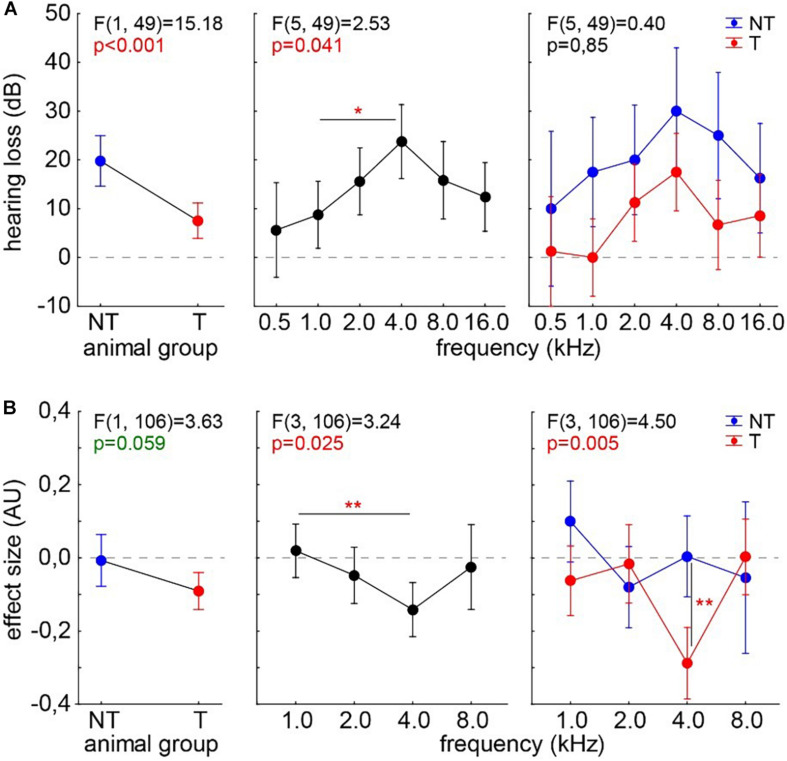
Analyses of hearing loss **(A)** and GPIAS effect size **(B)** in 16 animals after an acoustic noise trauma centered around 2 kHz. The animals are separated into those with behavioral signs of tinnitus (T, *n* = 10, red symbols) and those without such behavioral indications (NT, *n* = 6, blue symbols). Given are the results of the two-factorial ANOVAs with factors *animal group* and *stimulation frequency*. Both analyses show a peak effect at 4 kHz. Asterisks indicate significant Tukey *post hoc* tests: *p < 0.05, **p < 0.01.

For comparison, we analyzed ABR and GPIAS data from 16 animals before and after mild acoustic 2-kHz trauma. Acute hearing loss (Figure 7A) and the effect size for tinnitus assessment (Figure 7B) were analyzed 1 week post trauma by two-factorial ANOVAs with the factors *tinnitus animal group* and *stimulation frequency*. We found not only a significant difference in HL and a strong trend in effect size between the animals with (T) and without tinnitus (NT) (Figures 7A,B, left panels) but also significant peaks at 4 kHz in both measurements (center panels). As already published in our recent papers, T animals showed better hearing thresholds compared with NT animals. Especially in the effect size interaction of both factors, the significantly negative values at 4 kHz (Tukey *post hoc* test, *p* = 0.003) show that the behavioral changes are frequency dependent only in T animals, that is, in animals with negative effect size changes.

#### Correlation of ABR and GPIAS Data in Salicylate and Trauma Animals

**FIGURE 8:**
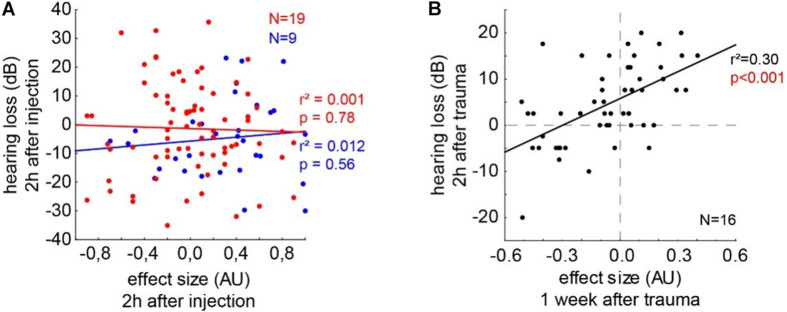
Correlations of the behavioral and electrophysiological data by multiple linear regression analyses. **(A)** The separate analyses for the animals in the C and S groups are depicted with their best linear fit for the correlation of effect size and HL after 2 h post-injection. Neither in the control group (*r*^2^ = 0.012, *p* = 0.56) nor in the salicylate group (*r*^2^ = 0.001, *p* = 0.78) a significant linear regression could be found. **(B)** Depicts the data of the 16 animals that received an acoustic trauma at 2 kHz with 115 dB SPL over 75 min. The correlation of the HL after 2 h post trauma and the subacute effect size measured after roughly 1 week shows a significant linear regression (*r*^2^ = 0.30, *p* < 0.001).

To test if the underlying neurophysiological mechanisms of salicylate and trauma-induced tinnitus are similar, we investigated the correlations of the behavioral and (far-field) electrophysiological data by multiple linear regression analyses (Figure 8). In the trauma-induced tinnitus model, we have already demonstrated that stronger tinnitus percepts, as indicated by more negative effect sizes in the GPIAS, are correlated with lower HT, which is a prediction of the model of the SR mechanism for tinnitus development (cf. Krauss et al., 2016). In Figure 8A, the separate analyses for the animals in the C and S groups are depicted with their best linear fit for the correlation of effect size and HL after 2 h post-injection. Neither in the control group (*r*^2^ = 0.012, *p* = 0.56) nor in the salicylate group (*r*^2^ = 0.001, *p* = 0.78), a significant linear regression could be found. Figure 8B depicts the data of the 16 animals that received an acoustic trauma at 2 kHz with 115 dB SPL over 75 min. The correlation of the HL after 2-h post trauma and the subacute effect size measured after roughly 1 week showed a significant linear regression (*r*^2^ = 0.30, *p* < 0.001), indicating that stronger tinnitus percepts (negative effect size values) lead to better HT (negative HL). In reverse conclusion, as the salicylate-induced tinnitus does not follow this pattern, it does not seem to rely on the same neurophysiological mechanism.

## Discussion

With this study, we aimed to investigate the neurophysiological mechanisms underlying salicylate and trauma-induced tinnitus. To this end, the effect of salicylate on hearing thresholds measured by ABR and behavioral correlates of a tinnitus percept assessed by GPIAS was tested in Mongolian gerbils, and the results were compared to the same variables in animals with noise trauma-induced tinnitus. For the latter, we hypothesize the underlying neurophysiological mechanism to be based on auditory information optimization processes based on an SR mechanism (cf. Krauss et al., 2016). We found that salicylate induced behavioral changes associated with a possible tinnitus percept. However, due to the observed shifts in hearing threshold and correlation analyses of tinnitus strength and HT, this percept is most probably not produced by an SR-induced increase of neuronal activity but must be based on a different neuronal mechanism.

The model of SR that has been recently proposed in our group (Krauss et al., 2016, 2019) predicts that HT should be improved within the frequency range of the tinnitus percept. In accordance with the SR model, the audiometric data of almost 40,000 patients from the ENT clinic in Erlangen, tinnitus patients had significantly better HT than patients without tinnitus in the low-frequency range up to about 3 kHz, that is, in the speech-relevant frequency range (Krauss et al., 2016, 2019). Additionally, utilizing this mechanism, we proposed a therapeutic approach to tinnitus suppression, using external acoustic noise to replace the internal neuronal noise. In a pilot study, this approach was successful in patients with a hearing loss not exceeding 40 dB (Ahlf et al., 2012).

In the present study, we could demonstrate in an animal model that salicylate-induced transient tinnitus is most probably based on a different mechanism. Salicylate has been shown to act on the outer hair cell (OHC) lateral wall stiffness (Lue and Brownell, 1999), increasing the membrane conductance of the OHCs (Stypulkowski, 1990) probably *via* acting on the voltage sensitivity of the motor protein prestin (Oliver et al., 2001; Grosh et al., 2004; Yu et al., 2008; Yang et al., 2009). Most likely, salicylate primarily influences electromotility and OHC non-linear capacitance *via* a direct interaction with prestin (Greeson and Raphael, 2009). We speculate that this mechanism provides an explanation for the hearing loss induced by salicylate, but the mechanism and the site of the generation of the tinnitus percept still remain unclear (Guitton et al., 2003). The effects of salicylate are not only limited to the periphery, as a direct central effect of salicylate has also been demonstrated (Basta et al., 2008; Chen et al., 2013). Indeed, salicylate can easily get through the blood–brain barrier (Jastreboff et al., 1986) and change the delicate balance between the excitatory and inhibitory circuits in the central auditory system (Xu et al., 2005). In fact, synaptic inhibition of the auditory cortex is predominantly GABAergic (Prieto et al., 1994a,b) and an alteration of these circuits can greatly change the response properties of auditory neurons (Rajan, 1998; Wang et al., 2000, 2002) and could consequently cause tinnitus (Eggermont and Roberts, 2004; Eggermont, 2005). Instead, noise trauma-induced tinnitus could be caused by central changes arising from the noise-induced reduction of cochlear input (Norena et al., 2002; Eggermont, 2007; Moffat et al., 2009). In other words, noise trauma-induced tinnitus has to have a central origin as well. However, we hypothesize that it develops due to an indirect effect triggered by damage in the cochlea and not due to a direct effect in the brain. One could speculate that the direct central effect of the salicylate might also contribute to the difference in the frequency range between the broad tinnitus percept induced by salicylate and the narrow phantom percept induced by sound exposure (Norena et al., 2010; Ahlf et al., 2012).

For assessing any change in hearing sensitivity, we used ABR measurements. In our data, the HT of the group C (Figure 2A) decreased over time in a frequency-independent manner. In that context, Ruebhausen and colleagues (Ruebhausen et al., 2012) noted that ABR generators were, primarily, in the central nervous system, and that interaction between general anesthesia and signal processing in the auditory brain stem would be expected. They conclude that, although both isoflurane and ketamine/xylazine were glutamatergic NMDA receptor antagonists, their global effect on neural systems would be complex and not known with sufficient precision to predict how each might affect auditory processing at a threshold. In our data, the HT before any kind of treatment (salicylate or saline) is comparable in groups C and S (Figure 3A). In group C, we see a reduction of HT over time, while, in group S, we do not observe this reduction, and we even find a frequency-dependent increase of the HT at 4 kHz 2 h post-injection (Figure 3B). This indicates that the effect of salicylate not only counteracts the reduction of HT but even increases the threshold in a frequency-specific manner. The reason why we see an effect only at 4 kHz is probably due to the effect of the salicylate in the OHC, which seems to be most prominent in the range of the best hearing. This means that the increase in the membrane conductance of the OHCs due to the effect of the salicylate would generate a stronger effect on the HT in the middle frequency region. Strikingly, this is not only a group effect (as demonstrated in the HT changed) but also an effect on an individual animal, as demonstrated by the HL at 4 kHz (Figures 4A,B). Why this effect is so specific in the frequency range of best hearing, one can only speculate. It may be due to basilar membrane thickness that peaks around this frequency range (Plassmann et al., 1987), making it more stiff and, therefore, reducing the effectiveness of the weaker pull from the affected OHCs further, as force production, along the cochlea, seems to be similar (Mahendrasingam et al., 2010) and also the numbers and innervation of these cells (Wilson et al., 1991) do not seem to change over the course of cochlear frequency locations.

For the assessment of a possible tinnitus percept, we used the behavioral approach of the GPIAS paradigm. GPIAS is the most common method for tinnitus assessment because it does not require any training, avoids conditioning-related plasticity, and saves time (Turner et al., 2006). However, it is still controversial if the method is appropriate for tinnitus screening, as the “filling-in” interpretation has been questioned (Campolo et al., 2013; Radziwon et al., 2015). Furthermore, a wide range of criteria for positive tinnitus detection has been used across different laboratories, and there, still, is no consensus on a “best practice” for statistical evaluation of GPIAS results, as it exists for other behavioral paradigms (Hinkle et al., 2003). The method has also been strongly criticized for being not reliable and does not rule out the possibility detecting hearing loss rather than tinnitus. In order to overcome these limitations, Schilling and coworkers (Schilling et al., 2017) developed a new statistical approach based on the effect size of the behavioral response, used as a normalized measure for the PPI change. The method is robust and does not require any removal of outliers [which, otherwise, is a common practice (Longenecker and Galazyuk, 2011)]. The negative values of the effect size are easy to interpret and indicate less effect of the gap relative to the response of the startle pulse after treatment, which is considered to indicate a “filling” of the gap by a tinnitus percept in that frequency range and cannot be appointed to hearing loss alone. The method is, among others, applicable for salicylate or mono- or binaural noise trauma-induced tinnitus studies. We, here, see a clear effect of the salicylate 2 h but not 20 min post-injection. This is supported by the results of Jastreboff and coworkers (Jastreboff et al., 1986), who found that following i.p. injection of salicylate, the maximum levels in blood serum occurred after 1.5 h, while the levels in the perilymph and spinal fluid reached their maximum within 2–4 h. Figures 5, 6 show the effect size, a normalized measure for the PPI change in the GPIAS (Hedges, 1982). If an animal has a stronger response to the gap (lower startle amplitude) during the post-recording compared to the precondition, the effect size will be positive. This may be due to proposed cortical learning effects that lead to increased responses to the startle (Moreno-Paublete et al., 2017). This phenomenon is always present, either when treating the animals with saline (Figure 5) or without treating the animals at all (unpublished data from our lab), or even when treating the animals with salicylate. On the other hand, the absolute values of the negative effect sizes can be interpreted as tinnitus severity, as it results from a smaller response to the gap, i.e., stronger startle amplitude due to potential “filling” of the gap by the tinnitus percept in the appropriate frequency range. The learning effects in the group S can be seen in those frequency ranges where tinnitus is not so strongly perceived (Figure 6B, 1 kHz) while negative effect sizes dominate the 4 kHz range. The consequence of the proposed cortical learning effect would be that the tinnitus percept must be strong enough to overcome the learning. In other words, we probably always underestimate the tinnitus percept, but, still, at 2 h post-injection, animals show a significant negative effect size compared to the control group at 4 kHz (Figure 6B). This matches perfectly with our data of HT shift, indicating that the salicylate-induced HL and the tinnitus percept both lie in the best hearing frequency range.

In data of trauma animals (Figure 7), we found clear effects of HL dependency on the effect size of the behavioral measurements, i.e., on a significant tinnitus percept in a least one frequency or the lack thereof. Interestingly, while the maximum HL was centered around the frequency range of best hearing, comparable with the effect of salicylate, salicylate-induced tinnitus increases HL while trauma-induced tinnitus decreases the effect of the trauma on hearing thresholds.

Comparable between both tinnitus induction methods is the maximum effect size change at exactly the frequency of best hearing/maximal HL in tinnitus animals only. This indicates that the behavioral outcome of both methods is comparable.

To further demonstrate that the underlying neurophysiological mechanism – independent if our hypothesis is correct or not – is different for trauma and salicylate-induced tinnitus, we correlated the effect size with the HL for all the given frequencies after 2 h post the injection in the groups C and S. As expected, in the group C, no correlation between the two variables can be found (Figure 8A, blue line) as, in this group, no tinnitus was induced. In the group S, we also found no correlation between the tinnitus strength and the HL (Figure 8A, red line). In contrast, in noise trauma-induced tinnitus, a significant positive correlation could be found, indicating that, for strongest tinnitus percepts (negative effect size), hearing thresholds are improved (negative HL). We can, therefore, conclude that, in noise trauma/hearing loss-induced tinnitus in rodents, the tinnitus percept is most probably based on the neurophysiological mechanism of SR, but salicylate-induced tinnitus is not based on that same mechanism. The exact neurophysiological differences between both models of tinnitus induction have to be investigated in further studies.

## Data Availability Statement

The raw data supporting the conclusions of this article will be made available by the authors, without undue reservation.

## Ethics Statement

The animal study was reviewed and approved by the Regierungspräsidium Unterfranken, Würzburg, Germany.

## Author Contributions

VL collected the data. VL and KT analyzed the data. VL, KT, and HS wrote the manuscript. All authors contributed to the article and approved the submitted version.

## Conflict of Interest

The authors declare that the research was conducted in the absence of any commercial or financial relationships that could be construed as a potential conflict of interest.

## Publisher’s Note

All claims expressed in this article are solely those of the authors and do not necessarily represent those of their affiliated organizations, or those of the publisher, the editors and the reviewers. Any product that may be evaluated in this article, or claim that may be made by its manufacturer, is not guaranteed or endorsed by the publisher.
