# Supplementary material for: Salicylate-Induced Changes in Hearing Thresholds in Mongolian Gerbils Are Correlated With Tinnitus Frequency but Not With Tinnitus Strength
Source: Front Behav Neurosci. 2021 Jul 30;15:698516. doi: 10.3389/fnbeh.2021.698516 (PMC8363116; doi:10.3389/fnbeh.2021.698516)
Supplement: Supplementary file 1 [file Table_1.DOCX]

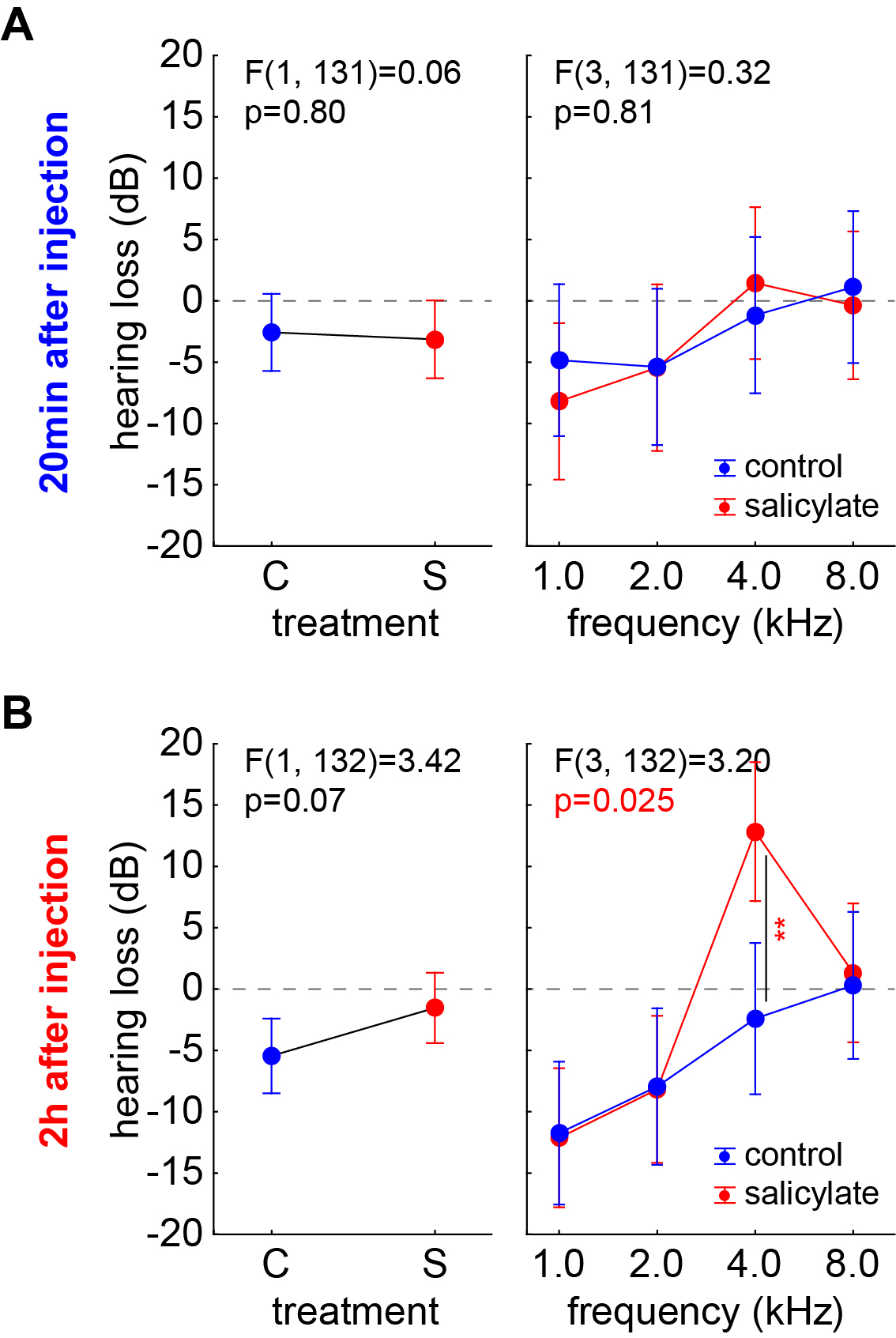


**Supplementary Figure 1**

Comparison of the HL of group C and S by for both time points independent two-factorial ANOVAs with the factors group (**left panels**) and its interaction with the *frequency* (**right panels**). **A:** 20 min after injection no significant effects of both factors on the HL could be identified**. B** 2h after injection no difference between both groups but a significant interaction of both factors with a significant Tukey post-hoc test at 4 kHz was detected.
